# Forgone care in patients with type 2 diabetes: a cross-sectional study

**DOI:** 10.1186/s12889-021-11639-2

**Published:** 2021-08-24

**Authors:** Habib Jalilian, Somayeh Heydari, Nazanin Mir, Saeedeh Fehresti, Rahim Khodayari-Zarnaq

**Affiliations:** 1 Department of Health Services Management, School of Public Health, Ahvaz Jundishapur University of Medical Sciences, Ahvaz, Iran; 2 Iranian Center of Excellence in Health Management, Department of Health Services Management, School of Management and Medical Informatics, Tabriz University of Medical Sciences, Tabriz, Iran; 3 Student Research Committee, School of Management and Medical Informatics, Iran University of Medical Sciences, Tehran, Iran; 4 Department of Health Economics and Management, School of Public Health, Tehran University of Medical Sciences, Tehran, Iran; 5 Department of Health Policy and Management, School of Management and Medical Informatics, Tabriz University of Medical Sciences, Tabriz, Iran; 6 Tabriz Health Services Management Research Center, Health Management and Safety Promotion Research Institute, Tabriz University of Medical Sciences, Tabriz, Iran

**Keywords:** Type 2 diabetes, Forgone care, Treatment withdrawal, Treatment discontinuation, Relinquished care, Delayed care

## Abstract

**Background and objective:**

Diabetes mellitus is a complex chronic disease requiring appropriate continuous medical care and delayed, or forgone care may exacerbate the severity of the disease. This study aimed to investigate the factors affecting forgone care in patients with type2 diabetes.

**Materials and methods:**

This was a cross-sectional study involving 1139 patients with type 2 diabetes aged> 18 years in 2019 in Tabriz, Iran. The researcher-made questionnaire was used for data collection. Data were analyzed using IBM SPSS software version 22 and IBM AMOS 22. Exploratory Factor Analysis (EFA) was performed for dimension reduction of the questionnaire, and Confirmatory Factor Analysis (CFA) used to verify the result of EFA. We applied the binary logistic regression model to assess the factors affecting forgone care.

**Results:**

Of the 1139 patients, 510 patients (45%) reported forgone care during the last year. The percentage of forgoing care was higher in patients without supplementary insurance coverage (*P* = 0.01), those with complications (P = 0.01) and those with a history of hospitalization (*P* = 0.006). The majority of patients (41.5%) reported that the most important reason for forgoing care is financial barriers resulting from disease treatment costs. Of the main four factors affecting, quality of care had the highest impact on forgone care at 61.28 (of 100), followed by accessibility (37.01 of 100), awareness and attitude towards disease (18.52 of 100) and social support (17.22 of 100).

**Conclusion:**

The results showed that, despite the implementation of the Islamic Republic of Iran on a fast-track to beating non-communicable diseases (IraPEN), a considerable number of patients with type2 diabetes had a history of forgoing care, and the most important reasons for forgoing care were related to the financial pressure and dissatisfaction with the quality of care. Therefore, not only more financial support programs should be carried out, but the quality of care should be improved.

**Supplementary Information:**

The online version contains supplementary material available at 10.1186/s12889-021-11639-2.

## Introduction

Type 2 Diabetes Mellitus is a complex chronic disease requiring appropriate continuous medical care [1]. Type 2 diabetes patients are often prescribed multiple medications to treat hyperglycemia, diabetes-related conditions like hypertension and dyslipidemia, and other comorbidities and complications. For those with diabetes, adherence to medications is associated with better control of intermediate risk factors [2–5], lower odds of hospitalization [4, 6–8], lower health care costs [6, 8–10], and lower mortality [4, 6]. On the other hand, access to equitable healthcare, pursuant to need, and regardless of demographic, ability to pay or social background, is an important goal for global healthcare systems [11]. Therefore, unmet need in health care systems is found as an undesirable feature [12].

Foregone care is one aspect of healthcare access; a person with forgone care needs is defined as one who does not use healthcare, despite perceiving a need for it. Forgone care is an important aspect in health system performance assessment as it shows a gap between the perceived need for health care and utilization. Further, forgone care is a highly important indicator for inequalities in access to health care. The consequences of forgone care are manifold such as “feeling worried” and/or problems with daily activities, higher use of emergency departments, the progression of the disease or result in the need for more complex treatment, lead to an increase in the utilization rate of health services in the future and finally, increase the health care expenditures [13–19]. The assessment of forgone care is mostly based on self-reports. Even though this is a subjective statement, it clearly shows considerable discomfort with the health care system, and it could also show a lost chance for improving the health status [20].

In the Iran health system, services are provided at three levels, namely: primary, secondary and tertiary. Only preventive and consultant services are delivered at primary health care, which is free of charge for patients. Services related to diabetes that are delivered at the primary care level are medicine (e.g. insulin and metformin) and basic technologies and procedures (e.g. blood glucose measurement, oral glucose tolerance test, HbA1c test, urine strips for glucose and ketone measurement) [21]. To other therapeutic services utilization, patients need to refer to secondary and tertiary healthcare providers, which are not free of charge. At these levels, a component of treatment costs is paid directly by the patients, and health insurance organizations pay the rest (e.g. the patient contribution for hospitalization services ranges from 10 to 30%, and for outpatient services ranges from 30 to 50%).

Given the increasing growth of non-communicable diseases and an increase in mortality rate due to these diseases in Iran, Iran’s Package of Essential Noncommunicable (IraPEN) disease as a part of the Health Sector Evolution Plan (HSEP), launched in 2014 by the Ministry of Health and Medical Education (MOHME), to provide universal health coverage, including access to NCD prevention and care and mental health services [21, 22]. The main focus of the HSEP plan was based on four main approaches: financial protection against catastrophic health expenditure, a decline in out-of-pocket payments (< 10% for inpatients and < 30% for outpatients), an increase in access to health services, and improvement in the quality of services [23–25]. In addition, given that IraPEN is developed based on the integrated and active care approach and evaluated through the control of parameters such as glucose, cholesterol and blood pressure, the evaluation of this program is essential in providing effective and continuous care to such patients at all care levels. So, a reduction in forgone care among patients with diabetes can be considered as one of the evaluation indicators of this program. The decision not to seek health care when one feels that care is needed (forgone care) is influenced by different factors [13]. Some of which may be amenable to policy intervention [26]. Therefore, the present study aimed to investigate the rate and causes of forgone care in patients with type 2 diabetes.

## Methods

### Study design

**Fig. 1:**
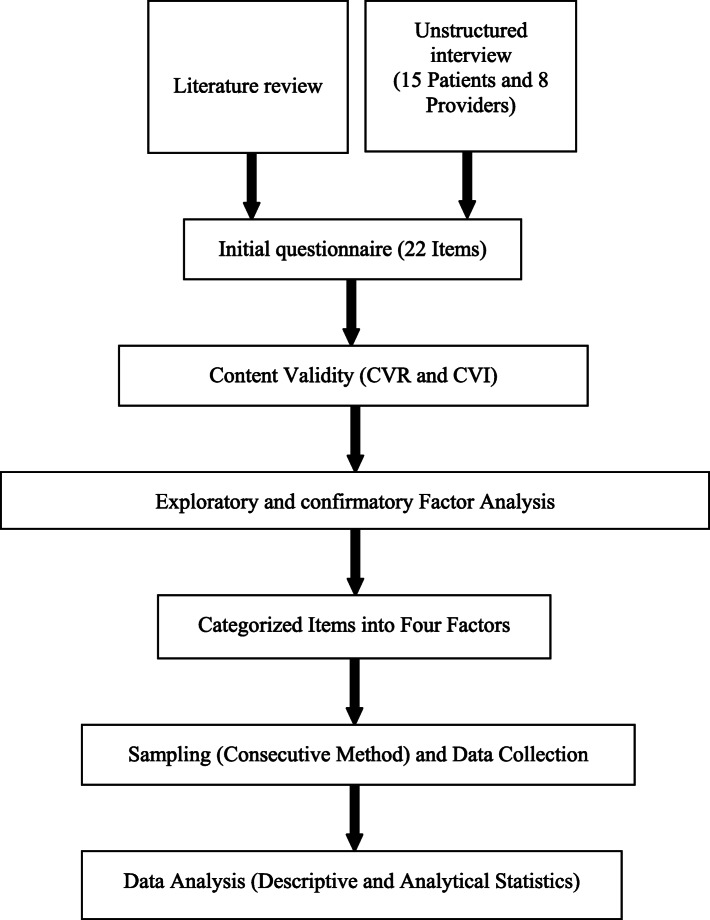
Methodological flowchart

A cross-sectional study was conducted on patients with type 2 diabetes in the East Azerbaijan province in 2019. The statistical population included all patients with type2 diabetes aged > 18 years without having physical and mental disabilities attending the educational hospitals (Imam Reza and Sina’s educational and therapeutic centers), public clinics (Asad Abadi and Sheykh Al-Raees), Endocrinologist office and primary healthcare centers in Tabriz, Iran (Fig. 1).

### Sample size and sampling methods

According to the prevalence rate of diabetes (8.5%) in East Azerbaijan province [27] and the population size of East Azerbaijan province (2016 census, 3,900,000 people), the total number of people with diabetes was estimated at 331,500. So the estimated sample size using Cochran’s Sample Size Formula (*n = Nt2pq/Nd2+t2pq*) was 384 patients. However, considering the impact of sample size on results of factor analysis (a larger sample will lead to more reliable results) and the impossibility of conducting random sampling, with the research team’s consensus, 1139 patients were selected using consecutive sampling methods. During a one-month timeframe, all patients attending the therapeutic and health centers were included. Comrey and Lee (1992) provided the following guidance in determining the adequacy of sample size: 100 = poor, 200 = fair, 300 = good, 500 = very good, 1000 or more = excellent [28]. In our study, respondents who answered “yes” to any of these questions were classified as having forgone care: 1) Those who did not use healthcare services, despite the perceived need for them, and 2) Those who refused to have a follow-up or to continue the treatment and to take medication despite a doctor’s prescription.

### Data collection tools

The researcher-made questionnaire was used to examine the factors affecting foregone care. The questionnaire consists of three parts; 1- demographic and socioeconomic variables, 2- variables related to disease (chronicity (in years) of DM, Type of current treatment, presence of co-morbidity or complications, the history of hospitalization due to complications of DM during the last year, disease severity) and 3- items related to reasons of forgoing care in type 2 diabetes patients. Those who were literate filled in the questionnaire, and those who were illiterate the questions were read to them, and they responded accordingly.

After reviewing the related literature, 15 interviews with type 2 diabetes patients and 8 interviews with doctors, nurses, and primary health care providers were conducted to develop the questionnaire. After reviewing the related literature and analyzing the interviews, 22 questions were extracted. In the next step, to assess the validity of the questionnaire, the questionnaire was given to 40 experts, including faculty members, specialist doctors, general practitioners, nurses and primary health care providers. Then, Content Validity Index (CVI) and content validity ratio (CVR) were computed using Microsoft Excel software for each question. Four questions did not meet the acceptable scores and removed from the questionnaire. In order to assess the reliability of the questionnaire, internal consistency and Cronbach’s alpha was used.

Furthermore, each question was rated on a 5-point Likert scale; (1-Very low importance, 2- Low importance, 3- moderately important, 4- more important and 5- much more important). For each dimension, the sum of the scores of the questions in each dimension was divided into the number of questions in each dimension, and by this way, the mean score for each dimension was calculated. For each dimension, higher scores indicate more impact forgone care.

The formula used for the calculation of the scores was as follows:

$$ \left(\mathrm{Obtained}\ \mathrm{score}\ \mathrm{in}\ \mathrm{subscale}-\mathrm{the}\ \mathrm{possible}\ \mathrm{lowest}\ \mathrm{score}\ \mathrm{of}\ \mathrm{subscale}\right)/\left(\mathrm{the}\ \mathrm{possible}\ \mathrm{highest}\ \mathrm{score}\ \mathrm{of}\ \mathrm{subscale}-\mathrm{the}\ \mathrm{possible}\ \mathrm{lowest}\ \mathrm{score}\ \mathrm{of}\ \mathrm{subscale}\right)\ast 100 $$

### Data analysis

Data analysis was performed using SPSS software version 22 and IBM AMOS 22. First, EFA (Principal Component Analysis extraction method) was used for dimension reduction. Then, the result of EFA tested by CFA. Descriptive statistics (frequency and percentage, mean and standard deviation) were used to assess the most common reasons for forgoing care and the importance of each factor affecting associated with forgone care. In the analytical section, the Chi-Square (K2) test applied to examine the association between the socio-economic and disease-related variables on the status of forgoing treatment. Also, T-test and one-way ANOVA were used to investigate the association between the socio-economic and disease-related variables and the total score of reasons associated with forgone care. A binary logistic regression model was applied to estimate the odds ratios for reported forgone care.

### The results of EFA and CFA

In this study, EFA was applied to ascertain latent variables. The Kaiser-Meyer-Olkin (KMO) and Bartlett test of sphericity were .869 and *P* = 0 < .0001, respectively, which was statistically acceptable. The results of EFA showed that the questions categorized into four dimensions (factors), including Quality of care (7 questions), Social support (4 questions), Awareness of and attitude towards disease and treatment (4 questions) and Accessibility (3 questions) (Table 1). The total loading of these factors was 60.53%. The share of quality of care, social support, awareness and attitude and accessibility factors was 27.50, 11.59, 11.41 and 10.02% of the variance, respectively. The content validity of the questionnaire was evaluated using content validity. The mean scores for the CVI and CVR were 0.89 and 0.63, respectively. The reliability of the questionnaire was evaluated via internal consistency, and Cronbach’s alpha was 0.83. The Cronbach alpha coefficient for quality of care, social support, awareness and attitude, and accessibility was .92, .76, .73 and .72, respectively.

**Table 1:** Rotated Component Matrix^a^

Questions	Items	Factor loading value
**Factor 1- Quality of care**		
**Q14**	I do not have enough trust in the skills, and scientific competence of the doctor	.898
**Q15**	I do not have enough trust in the service providers	.883
**Q5**	I am not satisfied with the quality of the provided care	.880
**Q11**	My doctor does not have a good relationship with me	.879
**Q6**	Health care provider deal with me violently and discriminately	.840
**Q18**	The behaviour of health care providers is unfair and discriminatory	.831
**Q4**	The waiting time for taking care is too long	.531
**Factor 2- Social support**		
**Q12**	I cannot communicate properly with my doctor	.682
**Q17**	My family does not support me in following and continuing the treatment process	.660
**Q16**	I do not have much desire to continue life	.624
**Q8**	I have a lot of family and working activities	.600
**Factor 3- Awareness and attitude**		
**Q9**	I’m unwilling to take medication	.796
**Q7**	I do not need the prescribed treatment	.776
**Q13**	I do not have enough awareness and knowledge of the consequences of forgone care	.680
**Q10**	I would prefer to use alternative therapies such as herbal medicines and traditional therapists	.446
**Factor 4- Accessibility**		
**Q1**	I do not have enough money to pay for the treatment of the disease	.686
**Q3**	The treatment process is too long and timely	.685
**Q2**	I have a long distance from the health care centres	.644

Extraction Method: Principal Component Analysis

Rotation Method: Varimax with Kaiser Normalization

^a^ Rotation converged in 6 iterations

**Fig. 2:**
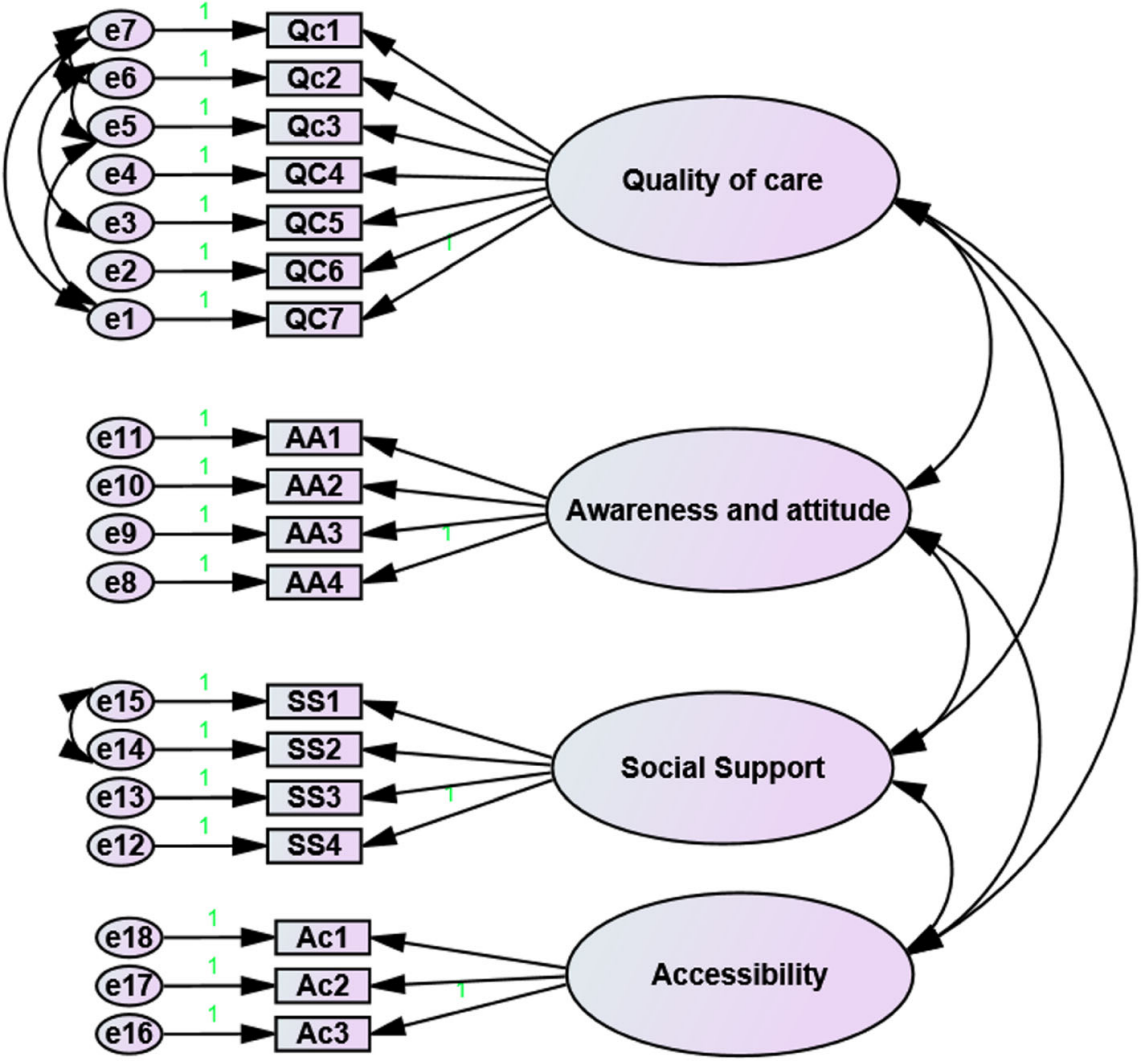
Research model

The results were verified again by EFA and CFA for illiterate and literate groups (Fig. 2). The results of Standardized Loadings for Confirmatory Model for illiterates and literates patients are shown in Table 2.The model fit and validity tests were also conducted. For both the literate and illiterate groups, the CFI (incremental fit indices) and GFI were > 0.9 and RMSE (absolute fit indices) were < 0.07. These results demonstrated the good fit of the model to the data (Table 3).

**Table 2:** Standardized Loadings for Confirmatory Model for illiterates (*n* = 473) and literates (*n* = 666)

			Illiterate			Literate		
			Estimate	S.E.	P	Estimate	S.E.	P
Ac3	<−--	Accessibility	.298			.412		
Ac2	<−--	Accessibility	.463	.389	< .0001	.639	.225	< .0001
Ac1	<−--	Accessibility	.641	.763	.001	.435	.210	< .0001
Qc7	<−--	Quality of care	.799			.753		
Qc6	<−--	Quality of care	.883	.052	< .0001	.882	.050	< .0001
Qc5	<−--	Quality of care	.905	.055	< .0001	.914	.052	< .0001
Qc4	<−--	Quality of care	.865	.056	< .0001	.913	.052	< .0001
Qc3	<−--	Quality of care	.778	.046	< .0001	.756	.043	< .0001
Qc2	<−--	Quality of care	.805	.054	< .0001	.847	.052	< .0001
Qc1	<−--	Quality of care	.418	.050	< .0001	.383		
Aa4	<−--	Awareness	.468			.725	.241	< .0001
Aa3	<−--	Awareness	.641	.181	< .0001	.471	.181	< .0001
Aa2	<−--	Awareness	.535	.165	< .0001	.665	.216	< .0001
Aa1	<−--	Awareness	.646	.178	< .0001	.671		
Ss4	<−--	Social support	.447			.756	.091	< .0001
Ss3	<−--	Social support	.441	.126	< .0001	.452	.074	< .0001
Ss2	<−--	Social support	.636	.206	< .0001	.257	.086	< .0001
Ss1	<−--	Social support	.690	.257	< .0001	.355	.044	< .0001
Quality of care	<−->	Awareness	.268	.041	< .0001	.180	.028	< .0001
Accessibility	<−->	Awareness				.351	.020	< .0001
Awareness	<−->	Social support	.589	.028	< .0001	.563	.028	< .0001
Quality of care	<−->	Social support	.478	.049	< .0001	.144	.039	.002
Accessibility	<−->	Social support				.477	.032	< .0001

**Table 3:** Goodness-of-Fit indicators for confirmatory factor analysis in illiterate and literate patients

Groups	GFI	CFI	RMSE	ECVI	Χ^**2**^/df
**Illiterate**	0.91	0.93	0.06	0.95	3.16
**Literate**	0.93	0.94	0.05	0.83	3.01

## Results

### Demographic and disease profile

A total of 1139 patients (389 from public hospital, 238 from public clinic, 214 from health centers and 298 from private clinic and Endocrinologist office) were included in the present study. Descriptive statistics for the study sample are shown in Table 4. Approximately two-thirds of patients were women (66.3%). Most patients (52.5%) had an income of < 1500 (international $) and were illiterate or only able to read and write (72.2%). Most people were treated with oral agents (54.3%) and insulin (39.4%), and only a small percentage of patients used behavior change. 72% responded they were aware of their disease for more than five years. More than two-thirds of the patients (76.1%) were afflicted with at least one of the complications of diabetes, and nearly one-third of patients (30.2%) were hospitalized during the last year due to diabetes complications. 58.7% of the patients reported that their disease was severe.

**Table 4:** Demographic and clinical profile and the frequency of forgone care

Variables	Categories	Frequency (Percent)	The history of forgone care %	ϰ^**2**^	***P-value***
**Gender**	Male	384 (33.7)	47.3	1.17	.27
	Female	755 (66.3)	43.9		
**Age**	< 40	127 (11.2)	50.8	3.19	.20
	40–60	527 (46.3)	46.0		
	> 60	485 (42.6)	42.4		
**Marital status**	Married	933 (81.9)	43.7	3.57	.059
	Single	206 (18.1)	51.0		
**Income status**	< 1500 international $	397 (52.5)	44.9	.36	.54
	> 1500 international $	359 (47.5)	42.7		
**Education status**	Illiterate	473 (41.5)	48.6	6.81	.07
	Reading and writing ability	407 (35.7)	44.4		
	Diploma	195 (17.1)	37.6		
	Academic education	64 (5.6)	44.4		
**Habitant status**	Urban	1003 (88.1)	45.2	.27	.60
	Rural	135 (11.9)	42.9		
**Type of basic health insurance**	Iranian health insurance	707 (64.4)	47.7	2.21	.13
	Social security	391 (35.6)	43.0		
**Supplementary health insurance status**	Yes	430 (39.1)	40.4	6.43	.01
	No	669 (60.9)	48.0		
**Chronicity (in years) of DM**	< 5 year	421 (37.1)	48.0	2.50	.28
	5 to 10 years	260 (22.9)	42.5		
	> 10 years	455 (40.1)	43.6		
**Type of current treatment**	Oral pills	619 (54.3)	45.8	.87	.64
	Insulin	449 (39.4)	44.7		
	Change in lifestyle (change in diet and physical activity)	71 (6.2)	40.0		
**Presence of co-morbidity or complications**	Yes	867 (76.1)	47.2	6.64	.01
	No	272 (23.9)	38.2		
**Hospitalization due to complications of DM in the past year**	Yes	344 (30.2)	51.2	7.50	.006
	No	795 (69.8)	42.4		
**Disease severity**	Sever	669 (58.7)	51.7	35.71	< .0001
	Moderate	232 (20.4)	29.6		
	Mild	238 (20.9)	41.2		

### The results of the rate of and the reasons for foregone care

Of the 1133 patients, 510 (45%) patients reported forgone care during the last year, which was higher in patients without supplementary insurance (P = 0.01). Moreover, forgone care was higher in patients with complications (*P* = 0.01), those with a history of hospitalization during the last year (P = 0.006) and those who rated their disease severity as very severe or very low compared to those who rated their disease severity as moderate (*P* < 0.001) Table 5.

**Table 5:** Total Variance Explained (Extraction Method: Principal Component Analysis)

Component	Initial Eigenvalues			Extraction Sums of Squared Loadings			Rotation Sums of Squared Loadings		
	Total	% of Variance	Cumulative %	Total	% of Variance	Cumulative %	Total	% of Variance	Cumulative %
**Q1**	5.322	29.569	29.569	5.322	29.569	29.569	4.952	**27.509**	27.509
**Q 2**	2.634	14.634	44.203	2.634	14.634	44.203	2.086	**11.590**	39.099
**Q 3**	1.672	9.290	53.493	1.672	9.290	53.493	2.054	**11.413**	50.512
**Q 4**	1.267	7.039	60.532	1.267	7.039	60.532	1.804	**10.020**	**60.532**
**Q 5**	.938	5.211	65.743						
**Q 6**	.824	4.576	70.319						
**Q 7**	.803	4.463	74.782						
**Q 8**	.690	3.831	78.613						
**Q 9**	.648	3.601	82.214						
**Q 10**	.576	3.198	85.412						
**Q 11**	.509	2.828	88.241						
**Q 12**	.480	2.669	90.909						
**Q 13**	.448	2.488	93.397						
**Q 14**	.358	1.990	95.387						
**Q 15**	.298	1.656	97.043						
**Q 16**	.228	1.269	98.312						
**Q 17**	.192	1.068	99.380						
**Q 18**	.112	.620	100.000						

The most frequently stated reason for forgoing care was financial burden resulting from the costs of disease treatment (41.5%), followed by disappointment and dissatisfaction with treatment outcomes (9.3%), lack of feeling the urge to medication and prescribed treatment (8.4), a long-distance from healthcare centers (8.0) and the long waiting time to receive services (6.4). Overall, 73.6% of patients reported these five items as the main reasons for forgoing care Table 6.

**Table 6:** The most common reasons to forgone care

Number	Causes	Frequency	Percent
**1**	**The financial burden due to the cost of treatment**	222	41.5
**2**	**Despair and dissatisfaction with the outcome of treatment**	50	9.3
**3**	**Lack of feeling the urge to medication and prescribed treatment**	45	8.4
**4**	**Long distances from the health care centers**	43	8.0
**5**	**Long waiting time and a long process of treatment**	34	6.4
**6**	**Dissatisfaction with the quality of provided care**	25	4.7
**7**	**Unwillingness to take medication**	25	4.7
**8**	**A lot of family and working activities**	22	4.1
**9**	**Use of alternative therapies such as herbal medicines and traditional therapists**	16	3.0
**10**	**Inadequate awareness and knowledge of the consequences of forgone treatment**	16	3.0
**11**	**The lack of family support for following the treatment process**	14	2.6
**12**	**Lack of trust in skills and scientific competence of the medical practitioner or the provider of the service**	12	2.2
**13**	**poor physician-patient relationship**	7	1.3
**14**	**Inappropriate behavior of health care providers (discriminatory behavior, violence and disrespectful behaviour)**	4	.7
**Total**		535	100.0

Of the four main factors which had an impact on forgone treatment, the highest mean score was related to the quality of care factor at 61.28 ± 39.74, followed by accessibility (37.01 ± 25.12), awareness and attitude towards disease (18.52 ± 18.87) and social support (17.22 ± 18.80).

The mean score of the *quality of care* factor was higher in men (*P* = 0.004), higher-income group (*P* < .0001), Iranian health insurance coverage groups (*P* = 0.003), those who had a longer duration of disease (*P* = .05), patients with complications (P < 0.001) and a history of hospitalization during the last year (*P* = .006) and those who rated their disease severity as severe (*P* < 0.001).

The mean score of *accessibility* factor was higher in low-income groups (*P* < .0001), patients with a lower level of education (*P* < 0.001), patients residing in rural areas (*P* < 0.001), those with Iranian health insurance coverage (*P* < .0001), those without supplementary insurance coverage (*P* < 0.001), patients with the history of hospitalization during the last year and those who rated their disease severity as severe (*P* < 0.001).

The mean score of *social support* factor was higher in younger patients (*P* = 0.001), unmarried patients (*P* = 0.01), patients with higher levels of education (*P* = 0.04), those with a history of hospitalization during the last year, and those who rated their disease severity as severe (*P* < 0.001).

Finally, the mean score of *awareness and attitude towards disease* factor was higher in younger patients (*P* = 0.02), unmarried patients (*P* = 0.01), those with Iranian health insurance coverage (*P* = 0.006), patients with a shorter duration of disease (*P* = 0.003) and those whose current treatment was a lifestyle change compared to those who take oral agents or inject insulin (*P* = 0.009) Table 7.

**Table 7:** Main factors affecting forgone care in terms of demographic, socioeconomic and disease-related variables

Variables	Categories	Social support		Awareness and attitude on disease and treatment		Quality of care		Accessibility	
		Mean ± SD	P-value	Mean ± SD	***P***-value	Mean ± SD	***P***-value	Mean ± SD	***P***-value
**Gender**	**Male**	18.11 ± 19.13	.26	19.24 ± 19.76	.37	66.04 ± 39.34	.004	37.59 ± 24.95	.57
	**Female**	16.77 ± 18.63		18.17 ± 18.40		58.87 ± 39.76		36.71 ± 25.22	
**Age**	**< 40**	22.83 ± 20.32	.001	21.89 ± 19.87	.02	61.07 ± 38.89	.98	41.14 ± 25.01	.08
	**40–60**	17.33 ± 19.73		19.07 ± 19.30		61.12 ± 39.55		37.31 ± 25.03	
	**> 60**	15.63 ± 17.02		17.05 ± 18.00		61.52 ± 40.25		35.59 ± 25.17	
**Marital status**	**Married**	16.57 ± 18.61	.01	17.88 ± 18.66	.01	61.53 ± 39.85	.65	36.45 ± 25.23	.10
	**Single**	20.19 ± 19.43		21.45 ± 19.59		60.18 ± 39.36		39.52 ± 24.52	
**Income status**	**< 1500 international $**	18.29 ± 21.01	.75	18.82 ± 19.94	.49	53.21 ± 38.56	< .0001	42.51 ± 25.96	< .0001
	**> 1500 international $**	17.83 ± 17.42		19.79 ± 18.85		68.13 ± 38.08		30.28 ± 23.59	
**Education status**	**Illiterate**	18.35 ± 19.48	.04	17.83 ± 17.95	.45	60.11 ± 39.26	.32	40.97 ± 25.00	< .0001
	**Reading and writing ability**	15.82 ± 17.89		18.41 ± 19.64		60.22 ± 40.44		37.19 ± 24.95	
	**Diploma**	16.02 ± 18.50		19.55 ± 19.18		64.26 ± 39.79		30.58 ± 23.60	
	**Academic education**	21.48 ± 19.50		21.28 ± 19.59		67.80 ± 38.57		26.30 ± 25.03	
**Habitant status**	**Urban**	16.83 ± 18.70	.057	18.15 ± 18.74	.07	61.39 ± 40.26	.81	35.18 ± 24.22	< .0001
	**Rural**	20.23 ± 19.39		21.40 ± 19.69		60.60 ± 36.00		50.49 ± 27.60	
**Type of basic health insurance**	**Iranian health insurance**	17.59 ± 17.94	.53	20.65 ± 18.83	.006	66.11 ± 38.82	.003	41.40 ± 25.96	< .0001
	**Social security**	16.87 ± 19.24		17.38 ± 18.83		58.72 ± 39.96		34.57 ± 24.17	
**Supplementary health insurance status**	**Yes**	16.40 ± 18.50	.23	18.48 ± 18.23	.94	63.72 ± 39.58	.09	30.74 ± 23.14	< .0001
	**No**	17.76 ± 18.99		18.55 ± 19.29		59.72 ± 39.80		41.09 ± 25.54	
**Chronicity (in years) of DM**	**< 5 year**	18.61 ± 20.12	.12	21.01 ± 20.96	.003	60.01 ± 39.44	.05	37.51 ± 24.52	.80
	**5 to 10 years**	15.68 ± 17.70		17.66 ± 17.33		57.24 ± 40.41		36.19 ± 23.53	
	**> 10 years**	16.88 ± 18.11		16.74 ± 17.45		64.47 ± 39.43		36.98 ± 26.60	
**Type of current treatment**	**Oral pills**	16.46 ± 18.85	.23	18.53 ± 19.07	.009	59.26 ± 40.68	.06	35.37 ± 25.09	.55
	**Insulin**	17.86 ± 18.32		17.50 ± 17.26		64.70 ± 38.61		38.96 ± 25.22	
	**Change in lifestyle (change in diet and physical activity)**	19.80 ± 21.18		24.91 ± 24.92		57.39 ± 37.47		39.04 ± 24.09	
**Presence of co-morbidity or complications**	**Yes**	17.38 ± 18.65	.63	18.35 ± 18.76	.57	64.50 ± 39.58	<.0001	37.74 ± 24.99	.08
	**No**	16.74 ± 19.30		19.09 ± 19.25		51.00 ± 38.57		34.66 ± 25.46	
**Hospitalization due to complications of DM in the past year**	**Yes**	19.07 ± 18.87	.03	18.36 ± 17.43	.84	66.03 ± 37.63	.006	40.86 ± 25.50	.001
	**No**	16.42 ± 18.73		18.60 ± 19.47		59.20 ± 40.48		35.34 ± 24.79	
**Disease severity**	**Sever**	19.09 ± 18.95	<.0001	19.57 ± 18.51	.083	65.99 ± 38.55	< .0001	41.29 ± 24.64	< .0001
	**Moderate**	14.79 ± 17.85		16.89 ± 17.91		46.17 ± 37.28		32.82 ± 24.10	
	**Low**	14.34 ± 18.73		17.19 ± 20.60		62.75 ± 41.92		29.02 ± 24.90	

Table 8 shows that factors of quality of care, accessibility, social support, awareness, and attitude on treatment and disease significantly affected foregone care in this population (P < 0.001). Accessibility and social support had the highest and lowest effect on forgone care, respectively.

**Table 8:** T-test results for main factors affecting forgone care

Variable	Forgone care	Mean ± SD	Mean Difference	t	P-value
**Quality of care**	No	57.78 ± 39.79	−7.99	−3.37	.001*
	Yes	65.77 ± 39.19			
**Accessibility**	No	31.53 ± 25.25	−12.41	−8.51	<.0001*
	Yes	43.95 ± 23.25			
**Social Support**	No	14.48 ± 16.58	−6.15	−5.53	<.0001*
	Yes	20.64 ± 20.75			
**Awareness and attitude on disease and treatment**	No	15.27 ± 16.66	−7.21	−6.52	<.0001*
	Yes	22.49 ± 20.53			

*P < 0.01 was considered as significant

The results of the binary logistic regression model are shown in Table 9. The Dependent variable “forgone care” was dichotomized into 0 = “no forgone care” and 1 = “reported forgone care”. The Hosmer–Lemeshow test results showed that the model is suitable (x^2^ = 7.57, *P* value = .47). The results indicate a significant association between forgone care and habitation status, disease severity, complications, quality, accessibility, as well as awareness and attitude. A 1% increase in the scores of awareness and attitude, accessibility and quality of care increases the odds of reporting forgone care by 2, 1 and 1%, respectively. Besides, the odds of reporting forgone care were 57% lower for patients with moderate disease severity than those with mild disease severity (P = 0.001). The odds of reporting forgone care were 49% greater for patients with complications than those without (P = 0.04).

**Table 9:** Binary Logistic Regression model for factors affecting forgone care

Variable	OR (95%CI)	p-value
**Complications (Reference = No)**		
**Yes**	1.49 (1.003–2.23)	0.04*
**Habitant status (Reference = Rural)**		
**Urban**	2.21 (1.25–3.94)	0.007*
**Disease severity (Reference = Mild)**		
**Sever**	1.08 (0.71–1.64)	0.71
**Moderate**	0.43 (0.26–0.72)	0.001*
**Accessibility**	1.01 (1.005–1.02)	0.001*
**Awareness**	1.02 (1.01–1.03)	0.003*
**Quality**	1.01 (1.001–1.02)	0.04*
**Social**	1.001 (1.008–1.02)	0.48

*P < 0.05 was considered as significant

## Discussion

This study aimed to examine the factors affecting forgone care among patients with type 2 diabetes. According to our results, nearly half of the patients reported forgone care, which was higher than in other studies. Röttger et al. conducted a study on patients with chronically ill in Germany. In their study, 14.1% of persons reported forgone care [29]. In the study of Towne SD Jr. BJ et al., among those with diabetes, the rate of forgoing care due to cost was 17.9% in 2011 and 14.7% in 2015, showing a slight decline [30]. Given the implementation of Iran’s Package of Essential Noncommunicable (IraPEN) disease, it can be concluded that the percentage of forgone care in patients with type2 diabetes is high, which is indicative of the poor performance of the healthcare system.

In this study, type 2 diabetes patients reported the financial burden resulting from treatment costs as the leading cause of forgoing treatment. Since most patients were elderly individuals with low socioeconomic status, and only a small percentage of patients were covered by supplementary insurance, the disease’s treatment cost was reported as the leading cause of forgone care. Bremer et al. in Germany showed that individuals with low income as well as people suffering from chronic illnesses face a higher financial burden and forgo health care services more frequently at the same time [31]. Kim et al. conducted a survey in 28 countries in 2017. They showed that income is significantly associated with forgone care in 21 of 28 examined countries, and people with lower income are more likely to forgo needed medical care [17]. A study indicated that difficulty paying medical bills increased the effect of lack of health insurance in predicting forgone medical care and had a conditional effect on the association between education and forgone prescription drug care [32]. Litwin et al. showed that forgone health care due to cost occurs among a substantial minority of older adults. Moreover, relinquished care is associated with younger old age, greater health needs and perceived economic inadequacy [33].

Frustration and dissatisfaction with the treatment outcomes reported as the second cause of forgone treatment. Given that diabetes and other chronic diseases require long-term care and the consequences of their treatment are not immediate and short-term, in many cases, the patient cannot make a reasonable association between receiving treatment and its outcomes. Hence, low quality of care and poor assessment of treatment efficacy are important factors in forgone treatment.

The third and most important factor affecting forgone treatment was related to the urge to prescribe medication and treatment. Of course, it seems that this factor was of high importance in patients with shorter disease duration and lower illness severity. Patients’ attitude towards disease plays an important role in patient adherence to prescribed medication. Whereby it is the duty of a physician and other providers to inform patients seriously about the consequences of irregular follow-up of treatment. The Fourth and fifth common cause of forgone treatment was related to a long distance from the health care centers and long waiting time to receive service. Long-distance from the health care centers was more likely to be common in patients residing in rural area. Long waiting time as one of the dimensions of the quality of care was alone one of the most common causes of forgone treatment. 73.6% of the patients reported these five factors as the most important reason to forgo treatment. Therefore, focusing on managing these five factors can greatly reduce the rate of forgone treatment and play a crucial role in better management of diabetes. Röttger et al. conducted a study on patients with chronically ill and indicated that forgone care could be influenced by different factors, on the system as well as individual level, which in the individual level, negative experiences (i.e. perceived discrimination) with health care are significantly associated with forgone care [29].

The rate of forgone care was higher in patients without supplemental insurance. For people who were not covered by supplementary insurance, financial barriers to access had a greater impact on their treatment withdrawal. Supplemental insurance can improve financial access to required services by paying basic health insurance franchise and reimbursement of the cost of services that are not covered by basic health insurance. Galbraith et al. showed that Membership in a High-Deductible Health Plan (HDHPs) and lower-income were independently associated with a higher probability of delayed/forgone care due to cost [34]. According to Reynolds et al., treatment discontinuation in patients with type2 diabetes was more in female, younger, Black or of Hispanic ethnicity, have more comorbidities, higher medication co-pays, start both OHAs together, have higher healthcare utilization before the index date and less likely to use prescription mail order in comparison with patients who did not discontinue [35]. In another study, adherence was independently associated with older age, male sex, a higher level of education and income, use of mail-order versus retail pharmacies, higher daily total pill burden, and lower out-of-pocket costs, and also patients who were new to diabetes therapy were less likely to be adherent [36]. Some other studies indicate an association between higher rates of forgone care and female sex, younger age, rural living, lack of health insurance, lack of financial support, low education levels, and poor health [13, 37–41].

In our study, forgone care was more likely to be higher among patients with complications and a history of hospitalization due to DM during the last year and those who rated their disease severity as very severe or very mild. These three variables somehow assess the severity of the disease. Since more complex and expensive services are needed among those with higher disease severity, the cost of care and the quality and effectiveness of treatment is highly important in these patients. Besides this, providing qualified and affordable services to these patients can reduce the withdrawal rate from these patients’ treatment.

Additionally, of the four main factors affecting forgone treatment, the quality of care had the highest impact on forgone treatment, followed by accessibility, awareness and attitudes towards disease and social support. This indicating the provided quality of care did not meet patients’ expectations. Although the mean score of the accessibility factor was lower than the quality of care factor, most patients were of the opinion that the main reason to forgo treatment is related to the financial burden resulting from the costs of disease treatment. Despite insurance coverage, diabetes imposes a considerable cost on patients with type2 diabetes, especially for lower-income patients. Therefore, in order to reduce the rate of withdrawal from treatment, it is necessary to provide financial support, such as strengthening insurance coverage and reducing copayment for low-income people to improve the financial access of these people to healthcare services. These results are in line with other studies in which forgone medical care was higher for those with lower incomes [16, 42, 43]. In Towne SD Jr. BJ et al. study, the rates of forgone medical care were higher among those with lower incomes (<$15,000; 24–31%) versus the highest (at/greater than $50,000; less than 10%), and higher for those with lower levels of education (without high school diploma/equivalent; above 20%) versus all other higher education categories (ranging from 9 to 18%) [30]. In summary, the impact of factors related to the healthcare system (health-care system based barriers) on forgone treatment was far more than factors related to the patient (patient-based barriers).

In this paper, the quality of the care factor’s impact was higher among patients with high income and education levels. In comparison, the impact of accessibility factor was higher in patients with low income and education levels. In other words, forgone care in low- income and high-income groups was more likely to be related to difficult financial access and poor care quality. In summary, the quality of provided care could not meet the expectation of patients with higher socioeconomic status, and the cost of provided care was unaffordable for those with lower socioeconomic status.

Based on this survey, the impact of the accessibility factor was higher in patients residing in rural areas than those residing in urban areas. People living in rural areas face more financial and physical barriers to receiving specialized and advanced services. Therefore, in order to promote equity in access to health care, it is necessary to take appropriate supportive measures to reduce these barriers in rural people. In the study of Towne SD Jr. BJ et al., the residents of rural areas with a diagnosis of diabetes had higher rates of forgone medical care (13–17%) than those in urban areas (11–15%) [30].

Dissatisfaction with the quality of care, accessibility barriers, poor awareness and attitude towards disease and treatment were likely to have a greater impact on the forgone treatment of patients covered by Iranian health insurance than those covered by social security insurance. Although the HSEP and IraPEN have been implemented exclusively in the Ministry of Health and its covered institutions, and Iranian Health Insurance is also covered by the Ministry of Health, the performance of the Social Security Organization has been better in this regard, and Social Security Organization tends to provide more cost coverage and high-quality service for its patients covered.

Younger patients were more likely to report having forgone care due to poor social support and poor awareness and attitude towards the disease than older ones. This difference may be because the disease is less severe in young people, and the complications of the disease have not yet appeared, so both patients and their families pay less attention to the control and management of their disease. Also, people with shorter disease duration are more likely to have poor awareness and attitude towards the disease, which can have a greater impact on forgoing care. Therefore, in order to prevent worsening of the condition of young people and those who have shorter disease duration, it is necessary to provide more social support and the required training on the risk of incidence of disease complications to these patients. In Towne SD Jr. BJ et al. study, forgone medical care was highest among those with lower age, with rates higher than 30% among those aged 18–24 for 2011 to 2013 [30].

The quality of care had a greater impact on forgoing care among patients with diabetes complications and a history of hospitalization because of diabetes complications, and those who rated their disease severity as very severe, as these people need more complex and advanced services and the quality of service is of importance regarding these services. Also, since these patients had lower social support, the probability of forgone care was higher among them. Also, access-related barriers have had a greater impact on forgone care in these patients, as disease status among these patients was more severe and required more complex and costly services. On the other hand, the financial burden caused by the treatment costs is considered as one of the most common causes of forgone care.

## Conclusions

The results showed that, despite the implementation of the Islamic Republic of Iran on a fast-track to beating non-communicable diseases (IraPEN), a considerable number of patients with type2 diabetes had a history of forgoing care, and the most important reasons for forgoing care were related to the financial barriers and dissatisfaction with the quality of care. Therefore, more financial support programs should be carried out, and the quality of provided care should be improved.

### Policy implication and recommendations

In order to achieve the goals of the HSEP and IraPEN and to have the best performance in managing diabetes, healthcare providers should improve service quality for type 2 diabetes. Besides, health insurance organizations should improve financial coverage and access to healthcare among the vulnerable populations (people with lower incomes and education and those residing in rural areas). Also, health care providers should categorize patients with type2 diabetes based on age, sex, income and education level, urban and rural areas, as well as disease severity level in addition to general strategies, and exclusive political interventions to be designed for each group. Determining patients’ copayment rate based on their income and ability to pay can be an effective strategy for reducing the forgone care in these patients. Also, focusing more on educational interventions in patients who are younger and have a shorter disease duration can be an effective strategy at the primary care level.

### Limitations

Since the list of all patients was not available, and access to all patients was impossible, random sampling was not possible, so a sequential sampling method was used in this study.

## Supplementary Information

**Additional file 1.**

**Additional file 2.**

## Data Availability

Data will be available upon reasonable request from the corresponding author.
