# Supplementary material for: Forgone care in patients with type 2 diabetes: a cross-sectional study
Source: BMC Public Health. 2021 Aug 24;21:1588. doi: 10.1186/s12889-021-11639-2 (PMC8386068; doi:10.1186/s12889-021-11639-2)
Supplement: Supplementary file 1 — Additional file 1. [file 12889_2021_11639_MOESM1_ESM.doc]

**The EFA results for all samples**

| **KMO and Bartlett's Test** | | |
| --- | --- | --- |
| Kaiser-Meyer-Olkin Measure of Sampling Adequacy. | | .869 |
| Bartlett's Test of Sphericity | Approx. Chi-Square | 9114.944 |
|  | df | 153 |
|  | Sig. | .000 |

| **Total Variance Explained** | | | | | | | | | |
| --- | --- | --- | --- | --- | --- | --- | --- | --- | --- |
| Component | Initial Eigenvalues | | | Extraction Sums of Squared Loadings | | | Rotation Sums of Squared Loadings | | |
|  | Total | % of Variance | Cumulative % | Total | % of Variance | Cumulative % | Total | % of Variance | Cumulative % |
| 1 | 5.322 | 29.569 | 29.569 | 5.322 | 29.569 | 29.569 | 4.952 | 27.509 | 27.509 |
| 2 | 2.634 | 14.634 | 44.203 | 2.634 | 14.634 | 44.203 | 2.086 | 11.590 | 39.099 |
| 3 | 1.672 | 9.290 | 53.493 | 1.672 | 9.290 | 53.493 | 2.054 | 11.413 | 50.512 |
| 4 | 1.267 | 7.039 | 60.532 | 1.267 | 7.039 | 60.532 | 1.804 | 10.020 | 60.532 |
| 5 | .938 | 5.211 | 65.743 |  |  |  |  |  |  |
| 6 | .824 | 4.576 | 70.319 |  |  |  |  |  |  |
| 7 | .803 | 4.463 | 74.782 |  |  |  |  |  |  |
| 8 | .690 | 3.831 | 78.613 |  |  |  |  |  |  |
| 9 | .648 | 3.601 | 82.214 |  |  |  |  |  |  |
| 10 | .576 | 3.198 | 85.412 |  |  |  |  |  |  |
| 11 | .509 | 2.828 | 88.241 |  |  |  |  |  |  |
| 12 | .480 | 2.669 | 90.909 |  |  |  |  |  |  |
| 13 | .448 | 2.488 | 93.397 |  |  |  |  |  |  |
| 14 | .358 | 1.990 | 95.387 |  |  |  |  |  |  |
| 15 | .298 | 1.656 | 97.043 |  |  |  |  |  |  |
| 16 | .228 | 1.269 | 98.312 |  |  |  |  |  |  |
| 17 | .192 | 1.068 | 99.380 |  |  |  |  |  |  |
| 18 | .112 | .620 | 100.000 |  |  |  |  |  |  |
| Extraction Method: Principal Component Analysis. | | | | | | | | | |

| **Rotated Component Matrix^a^** | | | | |
| --- | --- | --- | --- | --- |
|  | Component | | | |
|  | 1 | 2 | 3 | 4 |
| Ac1 |  |  |  | .686 |
| Ac2 |  |  |  | .644 |
| Ac3 |  |  |  | .685 |
| Qc1 | .531 |  |  | .501 |
| Qc2 | .880 |  |  |  |
| Qc3 | .840 |  |  |  |
| Qc4 | .879 |  |  |  |
| Qc5 | .898 |  |  |  |
| Qc6 | .883 |  |  |  |
| Qc7 | .831 |  |  |  |
| Aa1 |  |  | .776 |  |
| Aa2 |  |  | .600 |  |
| Aa3 |  |  | .796 |  |
| Aa4 |  |  | .446 |  |
| Ss1 |  | .682 |  |  |
| Ss2 |  | .680 |  |  |
| Ss3 |  | .624 |  |  |
| Ss4 |  | .660 |  |  |
| Extraction Method: Principal Component Analysis.  Rotation Method: Varimax with Kaiser Normalization. | | | | |
| a. Rotation converged in 6 iterations. | | | | |

**The EFA results for literate patients**

| **KMO and Bartlett's Test** | | |
| --- | --- | --- |
| Kaiser-Meyer-Olkin Measure of Sampling Adequacy. | | .858 |
| Bartlett's Test of Sphericity | Approx. Chi-Square | 5492.093 |
|  | df | 153 |
|  | Sig. | .000 |

| **Total Variance Explained** | | | | | | | | | |
| --- | --- | --- | --- | --- | --- | --- | --- | --- | --- |
| Component | Initial Eigenvalues | | | Extraction Sums of Squared Loadings | | | Rotation Sums of Squared Loadings | | |
|  | Total | % of Variance | Cumulative % | Total | % of Variance | Cumulative % | Total | % of Variance | Cumulative % |
| 1 | 5.192 | 28.844 | 28.844 | 5.192 | 28.844 | 28.844 | 4.951 | 27.503 | 27.503 |
| 2 | 2.894 | 16.080 | 44.924 | 2.894 | 16.080 | 44.924 | 2.115 | 11.748 | 39.252 |
| 3 | 1.655 | 9.197 | 54.121 | 1.655 | 9.197 | 54.121 | 2.018 | 11.209 | 50.461 |
| 4 | 1.188 | 6.602 | 60.722 | 1.188 | 6.602 | 60.722 | 1.847 | 10.261 | 60.722 |
| 5 | .940 | 5.224 | 65.946 |  |  |  |  |  |  |
| 6 | .829 | 4.607 | 70.553 |  |  |  |  |  |  |
| 7 | .826 | 4.588 | 75.141 |  |  |  |  |  |  |
| 8 | .708 | 3.935 | 79.076 |  |  |  |  |  |  |
| 9 | .659 | 3.663 | 82.740 |  |  |  |  |  |  |
| 10 | .567 | 3.150 | 85.889 |  |  |  |  |  |  |
| 11 | .512 | 2.843 | 88.732 |  |  |  |  |  |  |
| 12 | .460 | 2.554 | 91.286 |  |  |  |  |  |  |
| 13 | .439 | 2.437 | 93.723 |  |  |  |  |  |  |
| 14 | .352 | 1.954 | 95.677 |  |  |  |  |  |  |
| 15 | .288 | 1.599 | 97.276 |  |  |  |  |  |  |
| 16 | .212 | 1.178 | 98.454 |  |  |  |  |  |  |
| 17 | .176 | .978 | 99.432 |  |  |  |  |  |  |
| 18 | .102 | .568 | 100.000 |  |  |  |  |  |  |
| Extraction Method: Principal Component Analysis. | | | | | | | | | |

| **Rotated Component Matrix^a^** | | | | |
| --- | --- | --- | --- | --- |
|  | Component | | | |
|  | 1 | 2 | 3 | 4 |
| Ac1 |  |  |  | .665 |
| Ac2 |  |  |  | .667 |
| Ac3 |  |  |  | .630 |
| Qc1 | .495 |  |  | .473 |
| Qc2 | .891 |  |  |  |
| Qc3 | .839 |  |  |  |
| Qc4 | .891 |  |  |  |
| Qc5 | .908 |  |  |  |
| Qc6 | .888 |  |  |  |
| Qc7 | .827 |  |  |  |
| Aa1 |  | .800 |  |  |
| Aa2 |  | .591 |  |  |
| Aa3 |  | .767 |  |  |
| Aa4 |  | .420 |  |  |
| Ss1 |  |  | .652 |  |
| Ss2 |  |  | .648 |  |
| Ss3 |  |  | .647 |  |
| Ss4 |  |  | .670 |  |
| Extraction Method: Principal Component Analysis.  Rotation Method: Varimax with Kaiser Normalization. | | | | |
| a. Rotation converged in 6 iterations. | | | | |

**The EFA results for illiterate patients**

| **KMO and Bartlett's Test** | | |
| --- | --- | --- |
| Kaiser-Meyer-Olkin Measure of Sampling Adequacy. | | .862 |
| Bartlett's Test of Sphericity | Approx. Chi-Square | 3820.364 |
|  | df | 153 |
|  | Sig. | .000 |

| **Total Variance Explained** | | | | | | | | | |
| --- | --- | --- | --- | --- | --- | --- | --- | --- | --- |
| Component | Initial Eigenvalues | | | Extraction Sums of Squared Loadings | | | Rotation Sums of Squared Loadings | | |
|  | Total | % of Variance | Cumulative % | Total | % of Variance | Cumulative % | Total | % of Variance | Cumulative % |
| 1 | 5.564 | 30.911 | 30.911 | 5.564 | 30.911 | 30.911 | 4.967 | 27.597 | 27.597 |
| 2 | 2.323 | 12.907 | 43.818 | 2.323 | 12.907 | 43.818 | 2.177 | 12.095 | 39.692 |
| 3 | 1.738 | 9.654 | 53.471 | 1.738 | 9.654 | 53.471 | 2.042 | 11.345 | 51.037 |
| 4 | 1.331 | 7.393 | 60.864 | 1.331 | 7.393 | 60.864 | 1.769 | 9.827 | 60.864 |
| 5 | .977 | 5.429 | 66.294 |  |  |  |  |  |  |
| 6 | .850 | 4.724 | 71.018 |  |  |  |  |  |  |
| 7 | .773 | 4.296 | 75.314 |  |  |  |  |  |  |
| 8 | .676 | 3.756 | 79.070 |  |  |  |  |  |  |
| 9 | .628 | 3.489 | 82.559 |  |  |  |  |  |  |
| 10 | .590 | 3.278 | 85.837 |  |  |  |  |  |  |
| 11 | .525 | 2.914 | 88.751 |  |  |  |  |  |  |
| 12 | .450 | 2.501 | 91.252 |  |  |  |  |  |  |
| 13 | .410 | 2.276 | 93.528 |  |  |  |  |  |  |
| 14 | .341 | 1.893 | 95.421 |  |  |  |  |  |  |
| 15 | .281 | 1.562 | 96.982 |  |  |  |  |  |  |
| 16 | .226 | 1.257 | 98.239 |  |  |  |  |  |  |
| 17 | .201 | 1.118 | 99.357 |  |  |  |  |  |  |
| 18 | .116 | .643 | 100.000 |  |  |  |  |  |  |
| Extraction Method: Principal Component Analysis. | | | | | | | | | |

| **Rotated Component Matrix^a^** | | | | |
| --- | --- | --- | --- | --- |
|  | Component | | | |
|  | 1 | 2 | 3 | 4 |
| Ac1 |  |  |  | .695 |
| Ac2 |  |  |  | .607 |
| Ac3 |  |  |  | .716 |
| Qc1 | .587 |  |  | .503 |
| Qc2 | .863 |  |  |  |
| Qc3 | .839 |  |  |  |
| Qc4 | .860 |  |  |  |
| Qc5 | .882 |  |  |  |
| Qc6 | .875 |  |  |  |
| Qc7 | .830 |  |  |  |
| Aa1 |  |  | .722 |  |
| Aa2 |  |  | .599 |  |
| Aa3 |  |  | .825 |  |
| Aa4 |  |  | .466 |  |
| Ss1 |  | .679 |  |  |
| Ss2 |  | .683 |  |  |
| Ss3 |  | .584 |  |  |
| Ss4 |  | .659 |  |  |
| Extraction Method: Principal Component Analysis.  Rotation Method: Varimax with Kaiser Normalization. | | | | |
| a. Rotation converged in 6 iterations. | | | | |
