# Supplementary material for: Forgone care in patients with type 2 diabetes: a cross-sectional study
Source: BMC Public Health. 2021 Aug 24;21:1588. doi: 10.1186/s12889-021-11639-2 (PMC8386068; doi:10.1186/s12889-021-11639-2)
Supplement: Supplementary file 2 — Additional file 2. [file 12889_2021_11639_MOESM2_ESM.doc]

**Forgone care in patients with type 2 diabetes: A cross-sectional study**

Habib Jalilian^1^, Somayeh Heydari^2^, Nazanin Mir^3^, Saeedeh Fehresti^4^, Rahim Khodayari-Zarnaq^5*^

1. *Department of Health Services Management, School of Public Health, Ahvaz Jundishapur University of Medical Sciences, Ahvaz, Iran. Email: jalilian.mg86@gmail.com*
2. *Iranian Center of Excellence in Health Management, Department of Health Services Management, School of Management and Medical Informatics, Tabriz University of Medical Sciences, Tabriz, Iran. Email:* [*saeedehheydari5@yahoo.com*](mailto:saeedehheydari5@yahoo.com)
3. *Student Research Committee, School of Management and Medical Informatics, Iran University of Medical Sciences, Tehran, Iran. Email:* [*nazanin.mir2015@gmail.com*](mailto:nazanin.mir2015@gmail.com)
4. *Department of Health Economics and Management, School of Public Health, Tehran University of Medical Sciences, Tehran, Iran. Email:* [*Sa.fehresti666@yahoo.com*](mailto:Sa.fehresti666@yahoo.com)
5. *Department of Health Policy and Management, School of Management and Medical Informatics, Tabriz University of Medical Sciences, Tabriz, Iran. Email:* [*rahimzarnagh@gmail.com*](mailto:rahimzarnagh@gmail.com)

*Corresponding author:* Rahim Khodayari-Zarnaq

*Email:* [*rahimzarnagh@gmail.com*](mailto:rahimzarnagh@gmail.com)

*ORCID ID: 0000-0003-1626-4505*

Cellphone: +989147864767

- KMO values between 0.8 and 1 indicate the sampling is adequate.

**Factor Analysis**

| **KMO and Bartlett's Test** | | |
| --- | --- | --- |
| **Kaiser-Meyer-Olkin Measure of Sampling Adequacy.** | | .869 |
| **Bartlett's Test of Sphericity** | **Approx. Chi-Square** | 9114.944 |
|  | **df** | 153 |
|  | **Sig.** | <.0001 |

| **Total Variance Explained** | | | | | | | | | |
| --- | --- | --- | --- | --- | --- | --- | --- | --- | --- |
| **Component** | **Initial Eigenvalues** | | | **Extraction Sums of Squared Loadings** | | | **Rotation Sums of Squared Loadings** | | |
|  | **Total** | **% of Variance** | **Cumulative %** | **Total** | **% of Variance** | **Cumulative %** | **Total** | **% of Variance** | **Cumulative %** |
| **Q1** | 5.322 | 29.569 | 29.569 | 5.322 | 29.569 | 29.569 | 4.952 | 27.509 | 27.509 |
| **Q 2** | 2.634 | 14.634 | 44.203 | 2.634 | 14.634 | 44.203 | 2.086 | 11.590 | 39.099 |
| **Q 3** | 1.672 | 9.290 | 53.493 | 1.672 | 9.290 | 53.493 | 2.054 | 11.413 | 50.512 |
| **Q 4** | 1.267 | 7.039 | 60.532 | 1.267 | 7.039 | 60.532 | 1.804 | 10.020 | 60.532 |
| **Q 5** | .938 | 5.211 | 65.743 |  |  |  |  |  |  |
| **Q 6** | .824 | 4.576 | 70.319 |  |  |  |  |  |  |
| **Q 7** | .803 | 4.463 | 74.782 |  |  |  |  |  |  |
| **Q 8** | .690 | 3.831 | 78.613 |  |  |  |  |  |  |
| **Q 9** | .648 | 3.601 | 82.214 |  |  |  |  |  |  |
| **Q 10** | .576 | 3.198 | 85.412 |  |  |  |  |  |  |
| **Q 11** | .509 | 2.828 | 88.241 |  |  |  |  |  |  |
| **Q 12** | .480 | 2.669 | 90.909 |  |  |  |  |  |  |
| **Q 13** | .448 | 2.488 | 93.397 |  |  |  |  |  |  |
| **Q 14** | .358 | 1.990 | 95.387 |  |  |  |  |  |  |
| **Q 15** | .298 | 1.656 | 97.043 |  |  |  |  |  |  |
| **Q 16** | .228 | 1.269 | 98.312 |  |  |  |  |  |  |
| **Q 17** | .192 | 1.068 | 99.380 |  |  |  |  |  |  |
| **Q 18** | .112 | .620 | 100.000 |  |  |  |  |  |  |
| **Extraction Method: Principal Component Analysis** | | | | | | | | | |

**Rotated Component Matrix^a^**

| **Questions** | **Items** | **Factor loading value** |
| --- | --- | --- |
| **Factor 1- Quality of care** | | |
| **Q14** | I do not have enough trust in the skills, and scientific competence of the doctor | .898 |
| **Q15** | I do not have enough trust in the service providers | .883 |
| **Q5** | I am not satisfied with the quality of the provided care | .880 |
| **Q11** | My doctor does not have a good relationship with me | .879 |
| **Q6** | Health care provider deal with me violently and discriminately | .840 |
| **Q18** | The behaviour of health care providers is unfair and discriminatory | .831 |
| **Q4** | The waiting time for taking care is too long | .531 |
| **Factor 2- Social support** | | |
| **Q12** | I cannot communicate properly with my doctor | .682 |
| **Q17** | My family does not support me in following and continuing the treatment process | .660 |
| **Q16** | I do not have much desire to continue life | .624 |
| **Q8** | I have a lot of family and working activities | .600 |
| **Factor 3- Awareness and attitude** | | |
| **Q9** | I’m unwilling to take medication | .796 |
| **Q7** | I do not need the prescribed treatment | .776 |
| **Q13** | I do not have enough awareness and knowledge of the consequences of forgone care | .680 |
| **Q10** | I would prefer to use alternative therapies such as herbal medicines and traditional therapists | .446 |
| **Factor 4- Accessibility** | | |
| **Q1** | I do not have enough money to pay for the treatment of the disease | .686 |
| **Q3** | The treatment process is too long and timely | .685 |
| **Q2** | I have a long distance from the health care centres | .644 |
| **Extraction Method: Principal Component Analysis.**  **Rotation Method: Varimax with Kaiser Normalization.**  **a. Rotation converged in 6 iterations.** | | |
